# Chronic Kidney Dysfunction Can Increase the Risk of Deep Vein Thrombosis after Total Hip and Knee Arthroplasty

**DOI:** 10.1155/2017/8260487

**Published:** 2017-05-22

**Authors:** Qiangqiang Li, Bingyang Dai, Yao Yao, Kai Song, Dongyang Chen, Qing Jiang

**Affiliations:** ^1^Department of Sports Medicine and Adult Reconstructive Surgery, Nanjing Drum Tower Hospital, Clinical College of Nanjing Medical University, Nanjing, China; ^2^Department of Sports Medicine and Adult Reconstructive Surgery, Nanjing Drum Tower Hospital Affiliated with the Medical School of Nanjing University, Nanjing, Jiangsu 210008, China; ^3^Laboratory for Bone and Joint Diseases, Model Animal Research Center, Nanjing University, Nanjing, Jiangsu 210061, China

## Abstract

**Background:**

Deep vein thrombosis (DVT) is one of the major complications of total joint arthroplasty (TJA). Chronic kidney dysfunction (CKD) has proven to promote a proinflammatory and prothrombotic state and is prevalent among patients undergoing TJA. The purpose of this study is to identify whether CKD increase the risk of DVT following TJA.

**Methods:**

In a retrospective study, 1274 patients who underwent primary TJA were studied. CKD is graded in 5 stages. Univariate and multivariate analysis were used to identify the association of CKD and its severity with postoperative DVT.

**Results:**

There were 1139 (89.4%) participants with normal kidney function, 103 (8.1%) with mildly decreased kidney function, and 32 (2.5%) with stage 3 and 4 CKD. A total of 244 patients (19.2%) were diagnosed with DVT. Sixty-four patients (5.0%) developed symptomatic DVT. Advanced age, female gender, malignancy, and eGFR showed significant association with total DVT. BMI, thrombosis history, malignancy, and eGFR were associated with symptomatic DVT. After adjusting for age, gender, BMI, and malignancy, eGFR was found to be related to both total and symptomatic DVT.

**Conclusions:**

CKD is an important risk factor for both total and symptomatic DVT following TJA. Postoperative prophylaxis should be made a priority in this population.

## 1. Introduction

Total joint arthroplasty (TJA) is an effective surgical procedure which can improve the quality of life and function of patients affected by end-stage arthritis of the hip or knee [1, 2]. Outcomes following TJA are generally favorable with low rates of complications. However, some complications have significant consequences, including early revision, infection, dislocation, venous thromboembolism (VTE), and death [2–4]. Among them, VTE is a major complication including deep vein thrombosis (DVT) and pulmonary embolism (PE). The risk factors of VTE had been well documented in previous studies, including surgery, cancer, hospitalization, immobilization, obesity, exogenous hormones, pregnancy, and the puerperium [5]. However, VTE cannot be effectively predicted on a combination of these risk factors. Hence, identification of more risk factors can facilitate the prediction of VTE.

Chronic kidney dysfunction (CKD) is a clinical situation with prevalence rising from 10% in 1994 to 13% in 2004 in the United States [6]. It is especially common among elderly patients undergoing TJA. The reported incidence of CKD patients who require TJA is 17%, which is higher than that of the general population [7]. CKD has proven to promote a proinflammatory and prothrombotic state in patients. Compared with individuals with normal kidney function, CKD patients are reported to have higher levels of D-dimer, C-reactive protein (CRP), and fibrinogen [8–10]. In addition, previous studies have reported hemostatic abnormalities such as enhanced platelet activation and aggregation and lower levels of antithrombin in CKD and nephrotic syndrome patients [11–13]. Herein it is vital to investigate whether CKD patients are more susceptible to developing postoperative DVT than those with normal kidney function. Several studies have demonstrated a 1.6–2-fold increase in the incidence of DVT when compared to control patients [14, 15]. However, to the best of our knowledge, there is still a lack of consensus on the increased risk of VTE in CKD patients undergoing TJA. Wattanakit et al. [15] reported that patients with stage 3/4 CKD had an almost twofold increased risk for VTE compared with those with normal kidney function. Similarly, Tan et al. [16] found that stage 3A and 3B CKD substantially increased the risk of VTE with an odds ratio of 1.95 and 4.17, respectively. However, Miric et al. [17] found the risk of DVT in CKD patients following TKA was not significant. Besides, few studies have specifically investigated the severity of VTE in addition to its incidence. Therefore, in the current study, we aimed to address these questions on the basis of a large cohort of patients undergoing TJA to investigate the relationship between CKD and the incidence of postoperative DVT and to further determine its influence on the severity of this complication. Our hypothesis was that CKD patients have a higher risk of developing both symptomatic and asymptomatic DVT following TJA compared with patients without CKD.

## 2. Materials and Methods

### 2.1. Participants and Measurements

**Figure 1:**
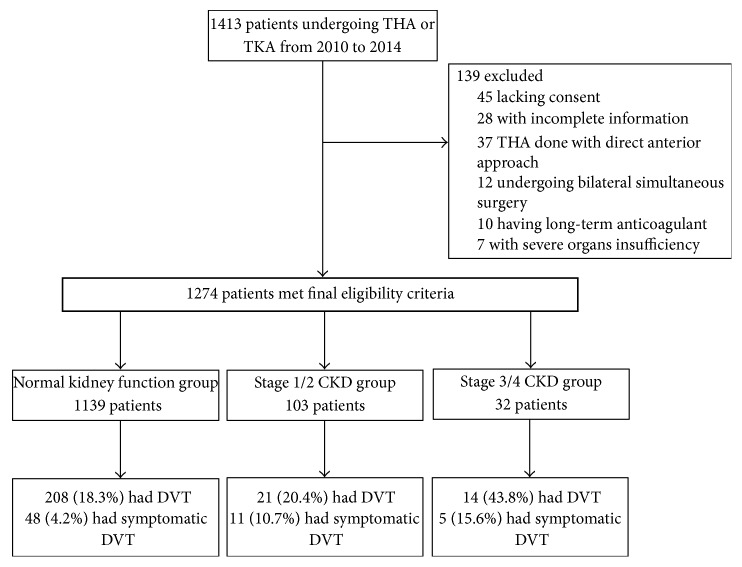
Flow diagram of study method. THA, total hip arthroplasty; TKA, total knee arthroplasty; DVT, deep vein thrombosis; CKD, chronic kidney function.

We conducted a retrospective analysis of the patients consecutively admitted for primary unilateral TKA and THA in our institution from October 2010 to August 2014. If patients received staged bilateral knee or hip surgeries during this period, only the first procedure was included. In addition, all THA were done using direct transgluteal approach. We excluded subjects with incomplete information, with severe organs insufficiency except kidney, or undergoing bilateral simultaneous surgery (Figure 1). The following baseline characteristics were collected, including age, gender, body mass index (BMI), diabetes, hypertension, malignancy, smoking history, thrombosis history, creatinine and blood urea nitrogen, D-dimer, C-reactive protein (CRP), and fibrinogen. The degree of renal dysfunction in CKD is graded in 5 stages. Normal kidney function was defined as estimated glomerular filtration rate (eGFR) ≥ 90 mL/min per 1.73 m^2^, mildly decreased kidney function as eGFR between 60 and 89 mL/min per 1.73 m^2^, and stage 3 to 4 CKD as eGFR between 15 and 59 mL/min per 1.73 m^2^. eGFR is calculated using creatinine measurement, along with age, sex, and race. The equation is based on the data of 454 Chinese patients with CKD as follows [18]:

(1) eGFR mL/min  per  1.73 m2=186×Pcr−1.154×age−0.203×1.223×0.742if  female.

Signed informed consent to venography was obtained from each participant before the procedure. Routine venography or color Doppler ultrasound following TJA was approved by the institutional review board.

### 2.2. Clinical Management and Assessment of DVT

All surgeries were performed by 2 experienced surgeons. All the patients received recommended thromboembolism prophylaxis regimen (low-molecular-weight heparin) and underwent unified rehabilitation program after surgery. All the patients received 0.3 mL (38 International Factor Xa Inhibitory Units per kilogram of body weight) of low-molecular-weight heparin from the same company subcutaneously once daily. The dosage level used as prophylaxis is considered to be moderate. In addition to drug thromboprophylaxis, bilateral lower limbs of all patients were compressed by pressure pump rhythmically to prevent DVT starting on the first postoperative night.

Routine venography of the operated lower limb was performed at 3–5 days after operation. For patients with contradictions to venography examination, color Doppler ultrasound was applied. Once confirmed by venography, color Doppler ultrasound, or symptoms of DVT, which is characterized by the presence of pain and swelling of the leg, skin discoloration, and a positive Homans' sign [19] or Neuhof's sign [20], conventional thrombolysis treatment was then performed. If DVT was not detected, patients would not receive any further anticoagulation treatment. All DVT was diagnosed by 2 experienced radiologists according to Robinov group's criterion [21]. Symptomatic DVT within 30 days after surgery was also recorded.

### 2.3. Statistical Analysis

Statistical analysis was performed using SPSS 19.0 system software (SPSS Inc., Chicago, IL, USA). Numeric data are shown as mean ± standard deviation; categorical data are shown as numbers with percentages. Qualitative variables including gender, hypertension, insulin resistance, smoking, malignancy, heart disease, and thrombosis history were compared by a chi-square test. Continuous variables including age, BMI, and DVT were analyzed by the Student *t*-test. The association of CKD with total DVT and symptomatic DVT was investigated using logistic regression analysis after adjustment for age, gender, BMI, malignancy, hypertension, diabetes, thrombosis history, and smoking by using logistic regression analysis.

## 3. Results

### 3.1. Demographic Characteristics

A total of 1274 subjects (500 TKA and 774 THA) were included in this study. There were 870 females and 404 males. The mean age was 65.2 ± 12.7 years (range: 18–93 years). The mean eGFR was 144.6 mL/min per 1.73 m^2^ (SD 52.3 mL/min per 1.73 m^2^). There were 1139 (89.4%) participants with normal kidney function, 103 (8.1%) with mildly decreased kidney function, and 32 (2.5%) with stage 3 and 4 CKD. The mean eGFR among the participants with stage 3/4 CKD was 43.0 mL/min per 1.73 m^2^ (SD 15.1 mL/min per 1.73 m^2^).

### 3.2. Incidence of DVT

Two hundred and forty-four patients (19.2%) with DVT were documented by venography or color Doppler ultrasound. Sixty-four patients (5.0%) developed symptomatic DVT within 30 days after surgery. There was no fatal pulmonary embolism (PE) as a result of the DVT. Patients with DVT had a greater mean baseline age, BMI, and higher prevalence of diabetes and hypertension than those without incident DVT.

### 3.3. eGFR and DVT

According to univariate analysis (Table 1), advanced age (*P* < 0.001), female gender (*P* = 0.007), malignancy (*P* = 0.046), and eGFR (*P* < 0.001) showed significant association with total DVT. BMI (*P* = 0.012), thrombosis history (*P* < 0.001), malignancy (*P* = 0.035), and eGFR (*P* < 0.001) were associated with symptomatic DVT. Malignancy (*P* = 0.046, *P* = 0.035) and eGFR (*P* < 0.001, *P* < 0.001) were related to both total DVT and symptomatic DVT. After adjusting for age, gender, BMI, and malignancy (Table 2), eGFR (OR = 1.36, *P* = 0.030; OR = 2.18, *P* < 0.001) was found to be related to both total DVT and symptomatic DVT.

As shown in Table 3, the incidence rates of total DVT and symptomatic DVT were 18.3% and 4.2% for normal kidney function, 20.4% and 10.7% for mildly decreased kidney function, and 43.8% and 15.6% for stage 3/4 CKD, respectively. In addition, the incidence rates of proximal DVT were 0.5%, 4.8%, and 14.3% for normal kidney function, mildly decreased kidney function, and stage 3/4 CKD, respectively. The OR for DVT with lower eGFR were similar for total DVT and symptomatic DVT. Compared with individuals with eGFR of 90 mL/min per 1.73 m^2^, the OR for total DVT for those with mildly decreased kidney function and stage 3/4 CKD patients were 1.03 and 2.68. The OR for symptomatic DVT for those with mildly decreased kidney function and stage 3/4 CKD patients were 2.82 and 3.55. For further examination of the relationship between kidney function and DVT, the predicted OR for DVT for those with eGFR of 75, 60, 45, and 30 mL/min per 1.73 m^2^ were 1.84, 2.68, 4.99, and 5.90, respectively, compared with individuals with eGFR of 90 mL/min per 1.73 m^2^.

## 4. Discussion

The aim of this study was to investigate the relationship between CKD and the incidence of postoperative DVT and to further determine its influence on the severity of this complication. We hypothesized that CKD patients have a higher risk of developing both symptomatic and asymptomatic DVT following TJA compared with patients without CKD. In this study, CKD was found to be an independent risk factor for DVT of patients undergoing TJA. Patients with stage 3/4 CKD had a 2.68-fold increased risk for DVT compared with those with normal kidney function, which was consistent with the data from previous studies. Using data of 19,000 middle-aged and elderly adults, Wattanakit et al. [15] reported that patients with stage 3/4 CKD had a relative risk of 2.09 for VTE as compared with subjects with normal kidney function. Similarly, Tan et al. [16] performed a retrospective study based on a cohort of 12,308 patients to identify if CKD influences the incidence of VTE after TJA. They found that stage 3A and 3B CKD substantially increased the risk of VTE with an odds ratio of 1.95 and 4.17, respectively. In addition, Shorr et al. [22] confirmed that those with stage 3B CKD had a higher risk of major VTE than those with moderate kidney impairment. Comparably, in our study the relative risk for VTE of the stage 3/4 CKD patients was 2.68, which was similar to the results reported by Wattanakit et al. [15] and Tan et al. [16]. To be noted, however, based on a retrospective analysis of 41852 primary TKA patients, Miric et al. [17] found the risk of DVT in CKD patients was not significant. Similarly, Warth et al. [7] demonstrated no significant differences of the risk of VTE between stage 4/5 CKD patients and patients without CKD. To our knowledge, both studies had recruited patients from various clinical centers without the unified diagnostic approach and criteria of postoperative DVT, which may lead to inaccurate and unreliable results. We believe our outcomes are reliable since we performed routine venography or color Doppler ultrasound in every single patient postoperatively, which is more accurate and sensitive in diagnosing early asymptomatic DVT. It is noteworthy that the results of the present study demonstrate that the risk of DVT was correlated with eGFR. Similar to the results in the present study, several studies have also demonstrated the relationship between the rate of VTE and eGFR [15, 16].

For the first time, this study also investigated the influence of CKD on the severity of the DVT. Proximal DVT including the popliteal vein, femoral vein, and iliac vein thrombosis has been reported to propagate more often than distal DVT [23], always accompanied by a higher PE risk [24]. In addition, compared with asymptomatic DVT, symptomatic DVT characterized by edema, redness, venous ectasia, and pain with calf compression can help give a clue of DVT for surgeons. In this study, we found that the odds ratio of symptomatic DVT together with the incidence of both symptomatic DVT and proximal DVT increased with the severity of CKD. It seems that patients with severe CKD may be more susceptible to the proximal DVT and symptomatic DVT.

The mechanism explaining the greater risk for VTE in CKD remains unclear. Hypercoagulability can be potentially induced by the increased inflammatory state associated with CKD. It was reported that the levels of various cytokines, such as interleukin-6, are elevated [10]. Adams et al. [25] reported that the protein C pathway was impaired in patients with CKD. Additionally, patients with CKD have higher levels of D-dimer, CRP, fibrinogen, factor VII, and factor VIII [8, 9]. Higher plasmin-antiplasmin complex with declining creatinine was identified, suggesting a state of reactive fibrinolysis [10]. Furthermore, as kidney function declines, levels of these inflammatory and procoagulant proteins climb. It is estimated that the elevated levels are due to increased synthesis out of proportion to urinary losses. Other hemostatic abnormalities have been reported in CKD and nephrotic syndrome patients including endothelial cell dysfunction, enhanced platelet activation and aggregation [11–13], activation of the coagulation system [26, 27], and decreased endogenous anticoagulants [27, 28]. In addition to CKD, surgery itself can act as a risk factor of VTE. Hence, it still requires further studies to evaluate the hemostatic mechanisms mediating the association between CKD and VTE.

Despite the fact that our data was derived from a large retrospective study, several limitations still existed in our study. First, we only tested early postoperative DVT by using venography or color Doppler ultrasound, which may therefore lead to missing cases with DVT after discharge. However, it has been reported that more than 80% of DVT after TJA occurs within 3 days postoperatively [29]. Moreover, once patients were diagnosed with negative DVT at early venography or color Doppler ultrasound in our study, they would receive aggressive rehabilitation program subsequently, which might have substantially reduced the risk of DVT. Second, we only performed venography or color Doppler ultrasound in the operated lower limbs. It was reported that DVT can be also detected in the nonoperated lower limb although the incidence is rare.

## 5. Conclusion

CKD can increase the risk of both total and symptomatic DVT after TJA. Our analysis suggests that postoperative prophylaxis should be made a priority in patients with CKD especially stage 3/4 CKD.

## Figures and Tables

**Table 1:** Characteristic data of the subjects.

Variables	Total patients			Total patients		
	Without DVT (*n* = 1030)	With DVT (*n* = 244)	*P* value	Without symptomatic DVT (*n* = 1210)	With symptomatic DVT (*n* = 64)	*P* value
Age (mean years ± SD)	64.4 ± 13.1	68.9 ± 10.1	<0.001	65.0 ± 12.8	67.9 ± 11.9	0.083
Female gender	686 (66.6%)	184 (82.1%)	0.007	820 (67.8)	50 (78.1%)	0.083
BMI (mean kg/m^2^ ± SD)	24.6 ± 4.2	25.0 ± 4.4	0.138	24.6 ± 4.3	26.0 ± 3.8	0.012
Hypertension	420 (40.9%)	107 (43.9%)	0.380	501 (41.4%)	26 (40.6%)	0.092
Insulin resistance	135 (13.0%)	27 (11.1%)	0.389	154 (12.7%)	8 (12.5%)	0.988
Smoking history	64 (6.2%)	14 (5.7%)	0.780	76 (6.3%)	2 (3.1%)	0.305
Malignancy	20 (2.0%)	10 (4.1%)	0.046	26 (2.1%)	4 (6.2%)	0.035
Heart disease	104 (10.3%)	26 (10.7%)	0.079	119 (9.8%)	11 (17.2%)	0.058
Thrombosis history	129 (12.5%)	38 (15.6%)	0.206	148 (12.2%)	19 (29.7%)	<0.001
eGFR (mean ml/min per 1.73 m^2^ ± SD)	148.1 ± 54.0	129.6 ± 40.9	<0.001	146.0 ± 52.5	116.5 ± 38.4	<0.001
D-dimer (mg/L)	1.3 ± 3.6	1.1 ± 2.2	0.590	1.2 ± 3.5	1.2 ± 2.0	0.970
CRP	4.9 ± 1.2	5.6 ± 0.9	0.073	5.1 ± 1.3	5.4 ± 1.0	0.154
Fibrinogen (g/L)	3.3 ± 0.8	3.2 ± 0.8	0.456	3.3 ± 0.8	3.3 ± 0.7	0.711

*P* < 0.05 was considered statistically significant.

TJA, total joint arthroplasty; DVT, deep vein thrombosis; SD, standard deviation; BMI, body mass index; eGFR, estimated glomerular filtration rate; CRP, C-reactive protein.

**Table 2:** Relationship between CKD and total and symptomatic DVT events in TJA after adjustment for age, gender, malignancy, and BMI.

Variables	Total DVT events		
	OR	95% CI	*P* value
Age	1.03	1.02–1.04	<0.001
Female gender	1.51	1.09–2.09	0.013
Malignancy	1.74	0.80–3.82	0.167
eGFR	1.36	1.03–1.81	0.030

Variables	Symptomatic DVT events		
	OR	95% CI	*P* value

BMI	1.07	1.01–1.13	0.016
Malignancy	0.35	0.12–1.08	0.068
Thrombosis history	2.71	1.53–4.80	<0.001
eGFR	2.18	1.44–3.32	<0.001

*P* < 0.05 was considered statistically significant.

CKD, chronic kidney dysfunction; eGFR, estimated glomerular filtration rate; DVT, deep vein thrombosis; BMI, body mass index; OR, odds ratio; CI, confidence interval.

**Table 3:** OR for total and symptomatic DVT by level of eGFR after adjustment for age, gender, malignancy, and BMI.

eGFR	Total DVT events				Symptomatic DVT events			
	Incidence	OR	95% CI	*P* value	Incidence	OR	95% CI	*P* value
>90^*∗*^	18.3%	1.00	N/A	N/A	4.2%	1.00	N/A	N/A
60–89	20.4%	1.03	0.62–1.71	0.920	10.7%	2.82	1.40–5.69	0.004
<60	43.8%	2.68	1.28–5.59	0.009	15.6%	3.55	1.27–9.93	0.016

^*∗*^Calculated as the reference for odds ratio.

*P* < 0.05 was considered statistically significant.

eGFR, estimated glomerular filtration rate; DVT, deep vein thrombosis; OR, odds ratio; CI, confidence interval; N/A, not applicable.

## Abbreviations

DVT: Deep vein thrombosis
VTE: Venous thromboembolism
PE: Pulmonary embolism
TJA: Total joint arthroplasty
TKA: Total knee arthroplasty
THA: Total hip arthroplasty
CKD: Chronic kidney dysfunction
eGFR: Estimated glomerular filtration rate
BMI: Body mass index
OR: Odds ratio
CRP: C-reactive protein.
